# Efficacy of Yushen Tongluo Granule Combined with Clomiphene Citrate for Anovulatory Infertility: A Double-Blind, Randomized, Placebo-Controlled Clinical Trial

**DOI:** 10.1155/2022/7933611

**Published:** 2022-01-27

**Authors:** Huayun Xu, Songpo Wang, Xiaohong Gao, Guozeng Wang

**Affiliations:** ^1^Department of Traditional Chinese Medicine, Shanghai General Hospital, Shanghai Jiao Tong University School of Medicine, Shanghai 200080, China; ^2^Department of Obstetrics and Gynecology, Shanghai General Hospital, Shanghai Jiao Tong University School of Medicine, Shanghai 200080, China; ^3^Department of Obstetrics and Gynecology, Shanghai East Hospital, Tongji University School of Medicine, Shanghai 200120, China

## Abstract

**Objective:**

To evaluate the efficacy and safety of Yushen Tongluo Granule (YSTLG) combined with clomiphene citrate (CC) in the treatment of anovulatory infertility.

**Methods:**

This randomized, double-blinded, placebo-controlled clinical trial was carried out in the Department of Obstetrics and Gynecology and the Department of Traditional Chinese Medicine (TCM). During the 3 menstrual cycle intervention periods, all subjects received 50 mg/day CC from day 5 until day 9 of the menstruation. If no ovulation, the amount of CC per cycle increased 50 mg/day until 150 mg/day. Participants in the experimental group received YSTLG, while participants in the control group received YSTLG placebo. The granules were orally taken from the end of menstruation until ovulation. When one leading follicle attained a diameter of 18 mm or more, 5000 U human chorionic gonadotropin (hCG) was given intramuscularly. The primary outcome measure was the ovulation rate, and follicular development was monitored by transvaginal ultrasound on the 10^th^ day of the cycles until ovulation. Secondary outcome measures including the overall curative effect, endometrial thickness, and pregnancy outcomes were also compared between the two groups.

**Results:**

The ovulation rate in the experimental group was higher than that in the control group (*P* < 0.05). Compared with the control group, the overall curative effect of the experimental group was better than that of the control group (*P* < 0.05), and the endometrial thickness in the ovulation phase was significantly thicker than that in the control group (*P* < 0.01). There was no significant difference in pregnancy rate and miscarriage rate between the experimental group and control group (*P* > 0.05).

**Conclusion:**

The combined YSTLG and CC used to treat anovulatory infertility can improve the ovulation rate without affecting endometrial thickness, which is efficacious and safe.

## 1. Introduction

Infertility is a group of reproductive disorders caused by a variety of causes. In China, 25% of couples actively attempting to become pregnant suffered infertility [1]. Infertility is a heavy burden on the family, especially for women, which can often cause severe anxiety and depression [2, 3]. Anovulation is an important cause of female infertility. It is found that 85% of anovulation was led to hypothalamic-pituitary-ovarian axis disorders caused by polycystic ovary syndrome (PCOS), abnormal body mass index (BMI), and endocrine diseases [4]. Therefore, restoring ovulation is an important link in the treatment of infertility.

As a nonsteroidal selective estrogen receptor regulator, CC, the first-line ovulation promoting drug, interferes with estrogen receptor in hypothalamus and blocks the negative feedback of estrogen on gonadotropin secretion, thus promoting follicular development [5]. However, due to its peripheral antiestrogen effect, CC is investigated to induce endometrial dysplasia and affect embryo implantation [6, 7]. Therefore, a novel therapy promoting ovulation without affecting the growth and development of endometrium is significant.

Traditional Chinese medicine has a long history in the treatment of infertility, which guaranteed a reliable outcome. The clinical data showed that traditional Chinese medicine had obvious characteristics and advantages in the treatment of ovulation induction on female infertility [8]. In addition, research studies also demonstrated that the performance of traditional Chinese medicine could regulate sex hormones to promote follicular development and improve endometrial receptivity in the treatment of ovulation disorder infertility [9, 10]. At the same time, some studies also find that Chinese herbal medicine may increase the effectiveness of CC therapy [11].

YSTLG contains pure, natural herbal ingredients; it is an empirical prescription developed by Professor Xiaosun Cai, a famous TCM physician. Until now, it has been successfully used in the clinical treatment for more than 30 years and has a significant therapeutic effect on anovulatory infertility. We speculated that YSTLG combined with CC in the treatment of anovulatory infertility may improve the ovulation rate without affecting the endometrial thickness during ovulation, so as to increase the pregnancy rate and reduce the abortion rate. Therefore, the goal of this trial was to evaluate the efficacy and safety of YSTLG combined with CC in the treatment of anovulatory infertility in a randomized, double-blind, placebo-controlled clinical trial.

## 2. Methods

### 2.1. Study Design

The study was a 3 menstrual cycle, double-blind, randomized, placebo-controlled clinical trial, conducted in the Department of Obstetrics and Gynecology and the Department of TCM of Shanghai General Hospital from August 2016 to July 2018.

This study was approved by the Ethics Committees of Shanghai General Hospital (grant no. (2017)09). Also, it was registered in the Chinese Clinical Trial Registry with the code number of ChiCTR2000040753 (https://www.chictr.org.cn/historyversionpuben.aspx?regno=ChiCTR2000040753). The study was carried out in accordance with the Declaration of Helsinki, and all eligible participants provided hand-written informed consent.

### 2.2. Drug Preparation

CC was obtained from Codal Synto Limited. YSTLG (12 g/bag) and YSTLG placebo (composed of 10% crude drug of YSTLG, dextrin, and stevia, 12 g/bag) were produced by the Shanghai Liantang Pharmaceutical Limited Company. The recipe in YSTLG consisted of Yinyanghuo (Herba Epimedii), Guizhi (Ramulus Cinnamomi), Bajitian (Radix Morindae Officinalis), Maidong (Radix Ophiopogonis), Dihuang (Radix Rehmanniae), Shudihuang (Radix Rehmanniae Preparata), Lulutong (Fructus Liquidambaris), Dingxiang (Flos Caryophylli), Shinanye (Folium Photiniae), and Huangjing (Rhizoma Polygonati). HCG was obtained from Shanghai First Biochemical Pharmaceutical Limited Company.

### 2.3. Diagnostic Criteria

#### 2.3.1. Diagnostic Criteria for Western Medicine

According to obstetrics and gynecology [12], Guiding Principles of Clinical Research on New Drugs of Traditional Chinese Medicine [13], the diagnostic criteria for anovulatory infertility are as follows: the inability to conceive after 1 year of unprotected intercourse of reasonable frequency, basal body temperature (BBT) charts showed no biphasic pattern for more than 3 months, vaginal smear showed no periodic change, cervical mucus crystallization showed no ellipsoid appearance, endometrial biopsy within 6 days of cycle showed no typical secretory phase change, and the level of progesterone was lower than that in luteal-phase. A diagnosis of anovulatory was made based on (1) and at least two of (2)–(6) were met.

#### 2.3.2. Criteria of TCM Differentiation of Symptoms and Signs

Kidney essence insufficiency pattern was diagnosed based on the Routine Diagnosis and Treatment of Traditional Chinese Medicine Syndrome in Shanghai [14] and National Standard for Clinical Terms of Traditional Chinese Medicine [15]. Primary symptom patterns included infertility following regular marital life for at least 1 year, delayed menstrual cycle and oligohypomenorrhea or amenorrhea, soreness, and weakness of waist and knees. Secondary symptom patterns included dizziness and tinnitus, sexual hypoactivity, amnesia, alopecia, fatigue, stringy, or deep and faint pulse. Diagnosis was made if 3 primary symptom patterns plus 2 secondary symptom patterns were identified.

### 2.4. Inclusion and Exclusion Criteria

Participants were adults aged 20–40 years old, who corresponded to the diagnosis criteria of anovulatory infertility and TCM syndrome differentiation. Eligible patients voluntarily joined the clinical observation and signed informed consent.

Exclusion criteria were as follows: infertility caused by congenital genitalia defects or malformations, infertility caused by genetic factors, infertility caused by endometriosis, adenomyosis, leiomyomas, and uterine dysplasia, infertility caused by tubal obstruction, immune infertility, infertility caused by male reproductive dysfunction, use of hormonal drugs (except for progesterone) or any other drugs which can affect reproductive endocrine in the past 3 months, complicated with severe cardiac, pulmonary, hepatic, renal, or neurological disease or mental illness, and complicated with thrombotic diseases.

### 2.5. Randomization and Blinding

Eligible participants were randomly assigned to groups A and B at a ratio of 1 : 1 according to the random number table. The table was generated through SPSS 18.0. Group A and group B corresponded to the experimental group (YSTLG) or control group (YSTLG placebo), respectively. YSTLG and YSTLG placebo had the same appearance, shape, color, and packaging, and the research physicians and participants were not able to know the difference.

### 2.6. Intervention

Both the experimental group and control group were given 50 mg/day CC for 5 days on the 5th day after the onset of spontaneous and progestin-induced menstruation as a basic treatment. If no ovulation, the amount of CC per cycle increased 50 mg/day until 150 mg/day. During the trial, participants in the experimental group were treated with YSTLG (12 g/bag, one bag per time, twice a day), while those in the control group were treated with YSTLG placebo (12 g/bag, one bag per time, twice a day). The granules were orally taken from the end of menstruation until ovulation. When one leading follicle attained a diameter of 18 mm or more, 5000 U hCG intramuscular injection was given. Timed intercourse was advised starting every other day for 1 week from the night of hCG administration. The treatment lasted for a total of 3 menstrual cycles, and drug administration was discontinued once the participant became pregnant during the trial.

### 2.7. Efficacy Criteria

According to the reference, clinical control (I): most of the clinical symptoms disappeared and the TCM syndrome total score decreased no less than 95%; significantly effective (II): most of the clinical symptoms disappeared and the TCM syndrome total score decreased no less than 70%; effective (III): some of the clinical symptoms improved and the TCM syndrome total score decreased no less than 30%; ineffective (IV): clinical symptoms did not improve or even deteriorate and the TCM syndrome total score decreased less than 30% [16].

### 2.8. Outcomes

#### 2.8.1. Primary Outcomes

Follicular development monitored by transvaginal ultrasound: transvaginal follicular monitoring was performed on all participants on days 10 and then individualized according to response. The ultrasound was performed once in 3 days when the diameter of the dominant follicle was less than 10 mm, every other day when 10–15 mm, and every day when more than 15 mm. Meanwhile, the maximum diameter of the dominant follicle during ovulation was recorded.

#### 2.8.2. Secondary Outcomes

(1) Endometrial lining monitored by transvaginal ultrasound: when one leading follicle attained a diameter of 15 mm or more, the endometrial thickness was recorded daily till ovulation by transvaginal endometrial lining monitoring. (2) Pregnant rate and miscarriage rate: a serum *β*-hCG concentration was determined 14 days after hCG injection if menses had not yet occurred. Biochemical pregnancy was defined as a falling *β*-hCG concentration on serial determination. Clinical pregnancy was diagnosed by visualization of a gestational sac with fetal heartbeat. Miscarriages were defined as biochemical pregnancies and/or cases with positive *β*-hCG testing who aborted spontaneously before reaching the stage of clinical pregnancy and/or cases aborting before 12 weeks of pregnancy.

### 2.9. Safety

General medical examination and regular blood tests, including blood routine and glutamic-pyruvic transaminase, glutamic-oxaloacetic transaminase, blood urea nitrogen, and creatinine, were examined before and after the trial treatment. All adverse events were recorded in the case report during the clinical trial. Additionally, the participants were provided with the researcher's telephone number and were instructed to call the researcher in case of possible side effects or deterioration of health.

### 2.10. Statistical Analysis

Statistical analysis was performed with the Statistical Package for the Social Science (SPSS Inc., Chicago, IL, USA) version 18.0. Quantitative data were summarized as mean ± standard deviation ($\bar{x}$ ± *s*). According to the normal distribution, the paired *t*-test was used for before and after treatment comparisons within the treatment and control groups, while the independent samples *t*-test was used between the two groups. To those who did not conform to normal distribution, the nonparametric test of rank transformation was used. Count data used case or percentage representation. The chi-square test was used for nonrank data and rank sum test for rank data. *P* < 0.05 was considered statistically significant.

## 3. Results

### 3.1. Patients Enrollment

**Figure 1:**
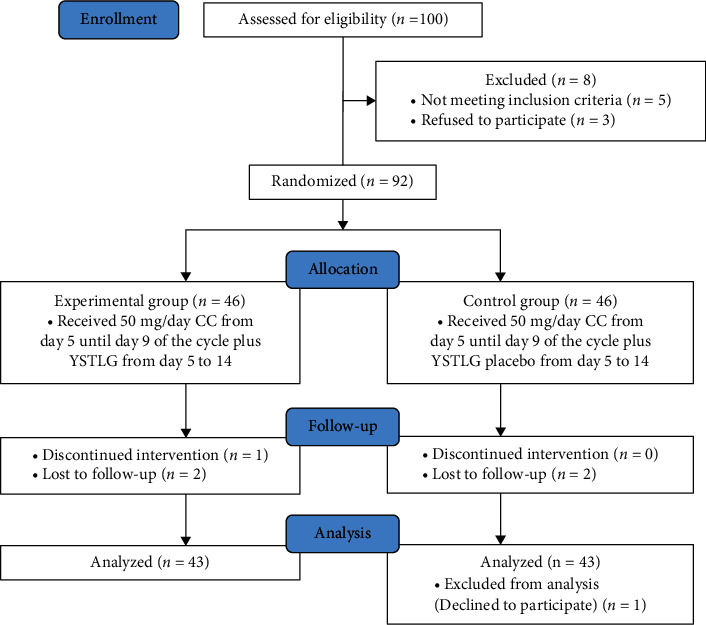
Flow diagram of patient enrollment, allocation, follow-up, and analysis.

As shown in Figure 1, of the 100 patients assessed for participation in the study, 5 did not meet the inclusion criteria and 3 refused to participate. A total of 92 recruited patients were allocated to each group (each group comprising 46 patients). During the study period, 4 of the 92 patients were lost. Of the 4 lost, 2 were from the experimental group and 2 were from the control group. Analysis included 43 patients in the experimental group and 43 patients in the control group.

### 3.2. Comparison of Baseline Characteristics between the Two Groups

Baseline demographic characteristics of all participants are given in Table 1. There was no significant difference in age, infertility duration, BMI, the number of PCOS, and reproductive history between the two groups (*P* > 0.05).

### 3.3. Primary Outcome Measures

As given in Table 2, the ovulation rate of the experimental group was higher than that of the control group, and the difference was statistically significant (*P* < 0.05).

### 3.4. Secondary Outcome Measures

#### 3.4.1. Comparison of Clinical Efficacy between the Two Groups after Treatment

As given in Table 3, the number of cured cases in the experimental group and the control group was 30 cases and 21 cases, respectively. Among them, 25 cases were pregnant in the experimental group and 21 cases in the control group. There was a significant difference in the overall efficacy between the two groups after treatment (*P* < 0.05).

#### 3.4.2. Comparison of Endometrial Thickness during Ovulation between the Two Groups

As given in Table 4, for nonpregnant women, endometrial thickness was monitored by continuous transvaginal ultrasound. The results showed that endometrial thickness increased significantly during the first, second, and third ovulation periods in the experimental group compared with the control group (*P* < 0.01).

#### 3.4.3. Comparison of Pregnancy Rate and Abortion Rate between the Two Groups

As given in Table 5, after treatment, more than half of the patients in the experimental group were pregnant, of which only 1 was aborted. There were 21 cases of pregnancy and 2 cases of abortion in the control group. There was no significant difference between the experimental group and the control group (*P* > 0.05).

### 3.5. Adverse Reactions

There were no adverse reactions observed in either group during the trial treatment period.

## 4. Discussion

One major strength is that this study is a randomized, double-blind, placebo-controlled clinical trial. For clinical trials, placebo-controlled trials can reliably demonstrate the efficacy of research drugs and can distinguish whether adverse events are caused by drugs. After 3 menstrual cycles of intervention, in this clinical trial, as ovulation rate was our primary outcome, the overall efficacy of the experimental group was better than that of the control group. Furthermore, the endometrium of 3 menstrual cycles ovulation of the experimental group was significantly thicker than that of the control group. We can conclude that the combined therapy with YSTLG and CC can improve ovulation rate and cannot affect the endometrial thickness during ovulation. Both of these are preconditions for a successful clinical pregnancy.

In addition, the number of cured and significantly effective cases in the experimental group is more than the control group, and there was a significant difference in the overall efficacy between the two groups after treatment. It showed that the overall efficacy of the combined therapy with YSTLG and CC was better than CC alone. However, there was no significant difference in the pregnancy rate and miscarriage rate between two groups (*P* > 0.05). Therefore, now, there was no evidence that the combined therapy can improve the pregnancy rate and reduce the abortion rate.

YSTLG has the effect of nourishing kidney and filling essence, promoting blood circulation, and dredging collaterals. Some studies found that the traditional Chinese medicine could increase the level of estradiol (E2) [17] and promote the blood supply of endometrium, induce angiogenesis, and improve endometrial receptivity significantly [18]. Besides, the drugs for promoting blood circulation and removing blood stasis can promote the proliferation of ovarian granulosa cells [19] and improve the blood supply to the uterus, resulting in increased blood flow of endometrium, hence promoting the growth and development of decidua [20]. A growing body of evidence supports that some herbal ingredients of YSTLG have estrogen-like effects, improve ovarian endocrine function, or promote angiogenesis. For example, it is found that estrogen-like substances in Herba Epimedii, which significantly increase the serum level of E2 [21], and icariin, an extract from Herba Epimedii, can thick the endometrium by increasing the expression levels of ER, VEGF, and KDR in endometrial cells [22] and improve the ovarian endocrine function [23]. Pharmacological studies show that Radix Rehmanniae Preparata has phytoestrogenic effects and could increase the immature rat's uterus wet weight and the ratio of uterus to body weight [24], and catalpol, an extract from Radix Rehmanniae Preparata, can improve both the quality and quantity of follicles on the sex organs [25]. It is also found that inulin-type oligosaccharides from Radix Morindae Officinalis roots promote angiogenesis by activating the PI3K-PKB-eNOS-signaling pathway [26]. Besides, Ramulus Cinnamomi and its active compounds can induce angiogenesis by upregulating the expression of VEGF in endothelial cells [27]. Therefore, we speculate that YSTLG may improve the ovulation rate and endometrial thickness by increasing the estrogen level or promoting endometrial angiogenesis, but the exact molecular mechanism remains unclear and requires further investigation.

This is the first time that a combination of YSTLG and CC has been studied for the treatment of anovulatory infertility. To date, only some studies have assessed the efficacy of traditional Chinese prescriptions for treating anovulation, and the quality of the evidence for other comparisons and outcomes was very low [28]. Similarly, nowadays, there is only limited evidence to suggest that the addition of Chinese herbal medicine to CC may improve pregnancy rates, but the quality of the evidence is low [28]. Our trial did not find that there were any significant differences between the experimental group and the control group in terms of pregnancy rate and miscarriage rate, which may be related to the small sample size, short intervention duration, and no postintervention follow-up. Moreover, in terms of safety, there were no adverse reactions observed between two groups during the trial treatment period.

In conclusion, the present study suggests that the combined treatment of YSTLG and CC used to treat anovulatory infertility is efficacious and safe, which can improve the ovulation rate without affecting endometrial thickness during ovulation, and make up for the defect of CC. The overall clinical effect of the combination therapy was better than that of CC alone. However, due to the small sample size, a confirmative conclusion is still premature. These findings could be used as preliminary data for further large-scale studies.

## Figures and Tables

**Table 1:** Baseline demographic characteristics of study participants.

Characteristics	Experimental group (*n* = 43)	Control group (*n* = 43)	*P* value
Age ($\bar{x}$ ± *s*, years)	30.67 ± 4.50	31.19 ± 4.58	0.603^*∗*^
Infertility duration ($\bar{x}$ ± *s*, years)	2.26 ± 1.02	2.50 ± 0.99	0.264^*∗*^
BMI ($\bar{x}$ ± *s*, years)	22.52 ± 1.36	22.83 ± 1.33	0.288^*∗*^
PCOS (*n* (%))	31 (72.09)	31 (72.09)	—
Reproductive history	—	—	—
Childbirth (*n* (%))	8 (18.60)	7 (16.28)	0.776^*∗∗*^
Spontaneous abortion (*n* (%))	18 (41.86)	12 (27.91)	0.175^*∗∗*^

^*∗*^Independent *t*-test. ^*∗∗*^Chi-square test.

**Table 2:** Comparison of ovulation rate between the two groups.

Group	*n*	Treatment cycles	Ovulation cycles (*n* (%))
Experimental	43	91	82 (90.10)
Control	43	99	78 (78.79)
*P* value	—	—	0.033

Values are reported as *n* (%) and analyzed by the chi-square test. Ovulation rate = ovulation cycles/treatment cycles × 100%.

**Table 3:** Comparison of clinical efficacy (*n* (%)).

Group	*n*	I	II	III	IV	Total effective, %
Experimental	43	30 (69.8)	5 (11.6)	6 (14.0)	2 (4.7)	95.3
Control	43	21 (48.8)	2 (4.7)	17 (39.5)	3 (7.0)	93.0

Analyzed by the nonparametric rank sum test, *Z* = 2.237, *P*=0.025. I, clinical control; II, significantly effective; III, effective; IV, ineffective.

**Table 4:** Comparison of endometrial thickness during ovulation between the two groups ($\bar{x}$ ± *s*).

Group	Endometrial thickness (1^st^ cycle) (mm)	Endometrial thickness (2^nd^ cycle) (mm)	Endometrial thickness (3^rd^ cycle) (mm)
Experimental	9.73 ± 2.11	9.38 ± 2.10	9.43 ± 2.29
Control	7.80 ± 2.25	7.22 ± 1.40	7.81 ± 1.72
*P* value	<0.001	<0.001	0.007

**Table 5:** Comparison of pregnancy rate and abortion rate between the two groups.

Group	Pregnancy cases (1^st^ cycle)	Pregnancy cases (2^nd^ cycle)	Pregnancy cases (3^rd^ cycle)	Total number of pregnancies	Abortions
Experimental	17	4	4	25 (58.1)	1 (4.0)
Control	14	2	5	21 (48.8)	2 (9.5)
*P* value	—	—	—	0.387	0.585

Values are reported as *n* (%) and analyzed by the chi-square test.

## Data Availability

The data used to support the findings of this study are available from the corresponding author upon request.
